# What Does the Dental Profession Most Need?

**Published:** 1906-06-15

**Authors:** Truman W. Brophy


### WHAT DOES THE DENTAL PROFESSION MOST NEED?

BY TRUMAN W. BROPHY, M.D., D.D.S., L.L.D.

We have men in our profession who compare favorably as scholars, mentally and scientifically and in the practice of our art, with the higher class of men in other professions, and yet I may truly say that one of the needs of the dental profession is the use and application of a broader general knowledge. The dentist's daily routine of work, which is such a draft upon his vital forces, rather tends to retard him from acquainting himself with the choicest literature of the day. Therefore, we need a greater number of men who will devote their time not only to familiarizing themselves with the current dental literature, but who will acquaint themselves also with the best standard writings of our leading authors. There is need in the dental profession of a higher appreciation of professionalism. It is indeed a sad comment on a class of men, who in many instances, appear to not have the slightest conception of what constitutes the difference between professional business and commercial business. The business man sells whatever his customers wish to buy —the want is made known and the article supplied. Too frequently the dentist follows the same practice, and instead of making use of his professional knowledge, and advising his patients as to the best course to pursue, he is often influenced to carry out his patients' wishes. The patient is frequently incompetent to express an opinion as to what the services to him should be. The physician, who is consulted by a patient, gives advice and directs the patient as to what is to be done and his work is usually accepted as law. The dentist should receive his patients in the same manner and advise them as to their needs, and in no instance be influenced to do that which he believes to be wrong to satisfy the whim of the patient, who is not able to judge of this needs.

I believe the long period of free services in the consultation room should end and that people who are able to pay for that which they receive should pay the dentist for his advice quite as freely as they would pay the physician for his. The dentist who has so little appreciation of the value of his time and professional advice that he does not exact a fee for it should mend his way. There is an ethical side of the shortcoming of a dentist who receives and advises his patients without fees. The tendency is to lower the standard of his profession among the people, and besides, he is not ethical to himself.

One of the greatest needs of the dentist is a more thorough knowledge of pathology and the closer observation of the phenomena presented in morbid conditions, also greater skill in performing the most difficult and complicated operations

Frequently, in my experience as an oral surgeon, patients are brought to me suffering from diseases of the mouth which have existed for a long period of time, and which have not been recognized by the medical or dental attendant. Therefore, the study of pathology and especially dental pathology, is essential. Many instances may be cited in illustration of the foregoing statement.

It is hard to impress upon the minds of students the value of the knowledge of chemistry. The dentist requires a more thorough understanding and knowledge of chemistry than the physician. He deals with nearly all the metals in his capacity as a dentist. Therefore, he should not only know their chemical, but also their physical properties. Chemistry, when thus considered and understood, is not a theoretical study; it is a practical one. The conditions which are active in bringing about the destruction of the dental organs will be understood only by thus acquainting one's self with the chemico-bacteriological changes. The dentist, therefore, needs a better knowledge of chemistry and its application to practice.

The dental profession needs a better knowledge and a closer application to details. Everything bearing on the dentist's work requires an application of exact knowledge. One part of his work which is defective may undo the usefulness of it all.

The teeth, more than any other organ of the body, are subject to disease. Nature often produces new tissue to restore other parts, which is quite the contrary in regard to the teeth. The teeth can not be brought back to usefulness except by the skill of a dentist, A medium good filling is a failure. There are only two kinds of fillings—good and bad—consequently too much stress cannot be laid upon manual training and the development of digital and manipulative skill. So one of the great needs of the dental profession is the higher development in our institutions of learning of manual training.

One of the great needs of the dental profession is a class of men who have been trained to teach, men who possess a broad knowledge and also who have become thoroughly educated in a school of dentistry, and who have studied pedagogics, the science and the art of teaching. The dental institutions need such men, who in addition, are eminently skilled in the departments of the dental college over which they preside. The time will surely come when the class of men engaged in teaching in our dental colleges will rank higher as teachers than they do to-day. The dental profession also needs a class of men to conduct the affairs of the profession in the capacity of dental examiners, men who have special qualities which fit them for their duties.

It is difficult under the present laws of the various States to secure the appointment of men whose professional, business and moral standing may always be relied upon to qualify them for the position, so long as dishonest men are able to hold political offices and use their influence in the appointment of men of their own class. The people at large and the profession are constantly menaced by the appointment of incompetent and sometimes disreputable dental examiners. The scandal that has arisen in some of our

States in connection with dental examining boards and the fact that bogus institutions have been issuing illegitimate diplomas through the years and that certain board members have been in consort with them and issuing licenses to men unqualified to receive them, brings prominently before us the necessity of doing everything within our power to insure the appointment of men of honor and ability to hold the important positions as examiners.

As I have previously stated, at a recent meeting of the Odontological Society of Chicago, I put the question to each of the twelve members present, "What does the dental profession most need?" At a following meeting, Dr. E. J. Perry stated that he had given the matter some consideration and had written upon the subject. It was my intention to quote from Dr. Perry's paper, but to do him justice I find it will be necessary to incorporate what he has written as follows:

"In responding to the question 'What does our profession most need at the present time,' I must reply to it out of the depths of my own needs. It is in my judgment mental industry, mental discipline; corporal bodily or physical industry, unaccompanied by mental effort, if such a thing is entirely possible, developes mental power, mind power and even spiritual peace. Grow strong by mental industry. Laziness of the mind, I believe, is one of our worst sins. We complain of a man who is physically lazy, but, I believe, mental laziness is much more prevalent, and infinitely more harmful. I don't believe any man who is lazy is truly happy. I believe that work is not only a blessing, but an absolute and indispensible necessity to true happiness. On the cover of the Philistine is the truism, 'The love you liberate in your work is the love you keep.' No man who does not love in the major sense is happy. And the underlying fundamental, actuating principle of it all is work, industry, both physical and mental. If a man would be an athelete he must work. He cannot satisfy his pleasures or indulge his appetite. He must be sanely and intelligently industrious, always doing the things he ought to do and resisting the things he ought not to do. If a man would be strong mentally he must not only be mentally industrious, but he must feed his mind on the right kind of food. Mental pastry is useless to the mind, he must resist his lazy desire to feed on it, he constantly needs to achieve a victory over himself, putting his mind at hard work, so acquiring a capacity for work, he must be a close student of himself.

"Dentists are mostly good fellows, but I believe they need above all else, *mental industry*. If we could but always do the things we ought to do, think the thoughts we should think, keep our minds fed with good mental food, read useful books, work out in the fresh air of good and useful thoughts, have as we can so easily, the companionship of the world's best minds, how different it would all be. How the world would light up to us, how many of our difficulties would melt away into easiness. *How* we *could* grow if we *would*. Is this not then our greatest need?

"My heart is sad when I look back and see how I have lost by a lack of mental industry. So many precious hours that they would add up into years have I lost, that are like the waters that have passed the mill without any purpose. My mental muscles are flabby, I am all bent over. I don't half fill my lungs with the good pure air. I keep in a little illy ventilated hut, when the great and blessed sunshine all about me is almost as free as God's gift of the atmosphere to men. I have just been too infernally lazy mentally, and God may never forgive me. My brother man gets but a part of what I owe him.

"While I may not be a type of the profession, I do feel that most of us need above all else outside of the things of the heart, mental industry, mental energy."

Members of the dental profession need a better understanding of business methods. The average dentist, like the average physician, is a poor business man. He should receive instructions in business methods and transact business in a business-like way. He should collect his bills monthly instead of permitting them to run on indefinitely and often lose them because he is not prompt in making collections. It is always easier to collect a bill within a month after service is rendered than it is in six months, besides people move, change locations and may become bankrupt, so that the man who is slow in collecting his bills often meets with a loss as a consequence.

A broader charity might be named as one of the needs of our profession. Oftentimes an injustice is done to a fellow practitioner in commenting upon the results of his efforts, when it would be impossible for the one thus criticising to know all the conditions and difficulties and obstacles accompanying the performance of the dental operation which has been adversely criticised. Character building or building professional renown has never been accomplished by attempting to pull others down. So, then, let us deal kindly with our brother even though he errs, and let us practice a broader charity.—*Review.*

